# The Potential of Peroxidases Extracted from the Spent Mushroom (*Flammulina velutipes)* Substrate Significantly Degrade Mycotoxin Deoxynivalenol

**DOI:** 10.3390/toxins13010072

**Published:** 2021-01-19

**Authors:** Ko-Hua Tso, Chompunut Lumsangkul, Jyh-Cherng Ju, Yang-Kwang Fan, Hsin-I Chiang

**Affiliations:** 1 Department of Animal Science, National Chung Hsing University, Taichung 40227, Taiwan; d100037004@mail.nchu.edu.tw; 2 Department of Animal and Aquatic Sciences, Faculty of Agriculture, Chiang Mai University, Chiang Mai 50200, Thailand; chompunut.lum@cmu.ac.th; 3 Graduate Institute of Biomedical Sciences, China Medical University, Taichung 40402, Taiwan; 4 Translational Medicine Research Center, China Medical University Hospital, Taichung 40402, Taiwan; 5 Department of Bioinformatics and Medical Engineering, College of Information and Electrical Engineering, Asia University, Taichung 41354, Taiwan

**Keywords:** deoxynivalenol, detoxification, *Fusarium graminearum*, lignin peroxidase, manganese peroxidase

## Abstract

Little is known about the degradability of mycotoxin deoxynivalenol (DON) by the spent mushroom substrate (SMS)-derived manganese peroxidase (MnP) and lignin peroxidase (LiP) and its potential. The present study investigated the growth inhibition of *Fusarium graminearum* KR1 and the degradation of DON by MnP and LiP extracted from SMS. The results from the 7-day treatment period showed that mycelium inhibition of *F. graminearum* KR1 by MnP and LiP were 23.7% and 74.7%, respectively. Deoxynivalenol production in the mycelium of *F. graminearum* KR1 was undetectable after treatment with 50 U/mL of MnP or LiP for 7 days. *N*-acetyl-D-glucosamine (GlcNAc) content and chitinase activity both increased in the hyphae of *F. graminearum* KR1 after treatment with MnP and LiP for 1, 3, and 6 h, respectively. At 12 h, only the LiP-treated group had higher chitinase activity and GlcNAc content than those of the control group (*p* < 0.05). However, more than 60% of DON degradabilities (0.5 mg/kg, 1 h) were observed under various pH values (2.5, 4.5, and 6.5) in both MnP (50 U/g) and LiP (50 U/g) groups, while DON degradability at 1 mg/kg was 85.5% after 50 U/g of LiP treatment for 7 h in simulated pig gastrointestinal tracts. Similarly, DON degradability at 5 mg/kg was 67.1% after LiP treatment for 4.5 h in simulated poultry gastrointestinal tracts. The present study demonstrated that SMS-extracted peroxidases, particularly LiP, could effectively degrade DON and inhibit the mycelium growth of *F. graminearum* KR1.

## 1. Introduction

During harvest, transportation, processing, and storage, feed ingredients are susceptible to fungal infection and contamination by their toxic metabolites—mycotoxins [1]. More than 25% of worldwide crops are affected by mycotoxins yearly, resulting in a huge financial loss in grain production and causing a threat to the health and life of humans and animals [2]. It is known that the 12,13-epoxide ring is the core chemical structure of trichothecenes [3]. Overall, the type B trichothecene deoxynivalenol (DON) is responsible for one of the most severe pollutions in feeds and ingredients. The primary DON toxigenic fungi are *Fusarium culmorum* and *Fusarium graminearum*, and the grains subjected to the highest intoxication rates from both fungi are wheat, barley, oats, and corn [4,5,6]. Also, global climate change has resulted in an increased risk of *Fusarium* infection in grains, and the worldwide pollution rates of DON in ingredients and feeds have been rising over years, posing a severe threat to animal and human health [7]. It has been reported that DON inhibits the syntheses of protein, DNA, and RNA, as well as the functions of mitochondrial membranes and eukaryotic cell division [8,9,10]. The DON limitations of the Food and Drug Administration (FDA) in the US and European Union (EU) are 1000 and 200–1750 μg/kg for human food and animal feed, respectively [11,12].

Strategies for mycotoxin degradation and detoxification include physical, chemical, and biological treatments. Physical treatment do not degrade DON in food and feed products effectively because of its high heat stability and low polarity [13,14]. Therefore, DON could remain stable in food and animal feeds for several years at room temperature or even with heat treatment over 100 °C [13]. In the feed industry, adsorbents such as montmorillonite and yeast cell walls are widely used to absorb mycotoxins in the gastrointestinal tract of animals [15]. However, mycotoxin adsorbents are effective only for high polarity mycotoxins such as aflatoxin B_1_ (AFB_1_); meanwhile, they may also adsorb feed nutrients, such as amino acids, water-soluble vitamins, and microminerals or likely trace minerals, making these nutrients and micronutrients unavailable for animals [16,17]. Chemical and biological degradations of DON may overcome some of these disadvantages because these detoxification methods could ameliorate the toxicity of DON by altering their chemical structures. However, chemical degradations to detoxify mycotoxins are not allowed according to European Union regulations for human food products [18] because they may alter the nutritional compositions of foods and feeds. Unlike chemical degradation, biological detoxification or degradation works through enzymatic degradation and only requires mild reaction conditions close to ambient temperature, which is practically applicable in the food and feed industries [19]. It mainly involves the use of microbes or enzymes to breakdown the toxic chemical structures of mycotoxins to lower the toxicity. Awad et al. [20] demonstrated that DON was degraded into de-epoxy-deoxynivalenol (DOM-1) through de-epoxidation by *Eubacterium* BBSH 797. For instance, many different bacteria, such as *Bacterial* LS100 and *Bacterial* S33, have been isolated from chicken intestinal microbes to convert DON into DOM-1 by de-epoxidation [21]. To date, several microorganisms from various sources such as soils, plants, and lake water, have been reported capable of degrading DON. For instance, the gram-positive bacterium *Nocardioides* sp. WSN05-2 isolated from wheat could degrade DON to 3-epi-deoxynivalenol by epimerization [22]. It was also found that the cytochrome P450 system in *Sphingomonas* sp. KSM1, a DON-utilizing bacterium isolated from lake water, was capable of catabolizing DON [23]. Moreover, studies have demonstrated that microorganisms and enzymes could degrade or conjugate with the chemical structure of mycotoxins to reduce their concentration and toxicity [24]. Given the 12,13-epoxide ring being the primary toxic moiety of DON [25], destruction of such key structure can be done by oxidative enzymes that catalyze epoxide ring-opening [26].

It is known that the main extracellular ligninolytic enzymes consist of lignin peroxidase (LiP), manganese peroxidase (MnP), and laccase. All these ligninolytic peroxidases are involved in lignin biodegradation [27]. Studies showed that the peroxidases of white-rot fungi can oxidize the benzene rings of phenols, non-phenolic aromatics, and amino-aromatic compounds into phenol oxides and hydroxyl free radicals via the epoxide ring-opening reactions [28]. To detoxify AFB_1_, MnP extracted from *Phanerochaete sordida* YK-624 can remove up to 86% of its original concentration in 48 h [29]. Previous studies have suggested that the 8,9-vinyl bond of AFB_1_ is first oxidized to 8,9-epoxide by MnP, and then being hydrolyzed to AFB_1_-8,9-dihydrodiol by adding the oxygen atom [29,30]. Some other studies indicated that MnP of white-rot fungi also effectively degraded DON [30,31]. Besides, not only MnP from white-rot fungi can degrade DON, but also other peroxidases from white-rot fungi or spent mushroom substrate (SMS) have a similar activity [32]. However, no report has detailed the degrading effects of other ligninolytic peroxidases and the antifungal abilities of both MnP and LiP. Therefore, we hypothesized that these peroxidases possess the potential for ring-opening reactions to achieve a real detoxification of DON. In the present study, the growth inhibition of *F. graminearum*, as well as DON degradability by MnP and LiP extracted from spent mushroom (*Flammulina velutipes*) substrate was demonstrated in vitro or in simulated gastrointestinal tracts.

## 2. Results

### 2.1. Effects of MnP and LiP on Growth Inhibition of F. graminearum KR1

The inhibition rates of *F. graminearum* KR1 mycelium after MnP and LiP treatments for 7 days were 23.7% and 74.7%, respectively (Table 1). However, the MnP treatment promoted (negative value of inhibition rate) slight mycelium growth instead of inhibition in the first 3 days during the experimental period. Beginning on Day 1 and continuing throughout the 7-day incubation period, the inhibition rate of *F. graminearum* KR1 with LiP treatment was significantly higher than with MnP treatment (*p* < 0.05).

### 2.2. Influence of MnP and LiP on Cell Wall Hydrolysis and Morphology

The chitinase activity and *N*-acetyl-D-glucosamine (GlcNAc) content of the cell wall were examined after the hyphae of *F. graminearum* KR1 was treated with MnP or LiP at 50 U/mL for various times (h). After treatment for 1, 3, and 6 h, the chitinase activity and GlcNAc content of the hyphae significantly increased in both treatment groups compared with that in the control group (*p* < 0.05), respectively (Table 2). At 12 h after treatment, only the LiP-treated group showed higher chitinase activity and GlcNAc content than those of the control treatment. Furthermore, the chitinase activity and GlcNAc content of the LiP-treated hyphae were significantly higher than those of the MnP-treated hyphae (*p* < 0.05), except for those at one hour after treatment (Table 2).

**Figure 1:**
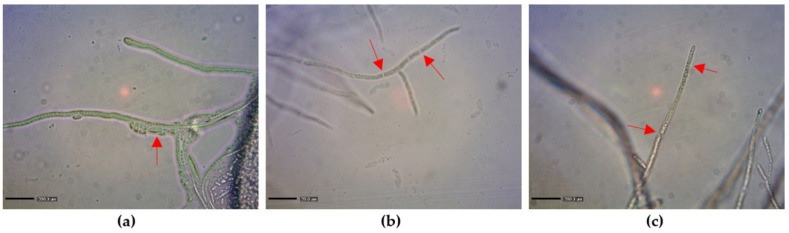
Morphological observations of *F. graminearum* KR1 hyphae after treatment with manganese peroxidase (MnP) or lignin peroxidase (LiP) for 12 h. (**a**) Treatment with the control solution shows intact hyphae with sporulation (arrow). (**b**) Treatment with 50 U/mL MnP shows broken hyphae with points of breakage (arrows). (**c**) Treatment with 50 U/mL LiP shows broken hyphae (arrows) as in the MnP treatment group. Bar = 200 μm.

The microscopic examination revealed that LiP treatment at 50 U/mL for 12 h induced marked changes in *F. graminearum* KR1 morphology. In treatments without MnP and LiP, *F. graminearum* KR1 mycelium demonstrated a dense cytoplasm (Figure 1a). With MnP treatment, some vesicles were visible inside the cell wall (Figure 1b). By contrast, large vesicles were observed inside the cell wall with LiP treatment (Figure 1c). Furthermore, LiP treatment at 50 U/mL led to significant collapse and breakdown of *F. graminearum* KR1 hyphae (Figure 1c) 12 h later.

### 2.3. Effects of MnP and LiP on DON Production of F. graminearum KR1

**Figure 2:**
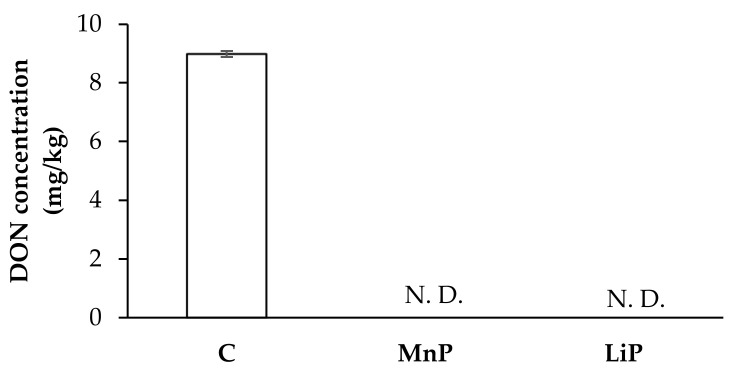
Deoxynivalenol (DON) production by *F. graminearum* KR1 grown in potato dextrose agar (PDA) for 7 days after SMS-extracted peroxidases treatments (50 U/mL). C: non-treated control; MnP: PDA with manganese peroxidase (MnP) treatment; LiP: PDA with lignin peroxidase (LiP) treatment. N. D.: not detectable (the limit of detection is 100 μg/kg for DON). Data are expressed as mean ± SD (*n* = 8).

Figure 2 depicts *F. graminearum* KR1 culture treated with MnP and LiP at 50 U/mL for 7 days: DON production was not detected in either treatments (the limit of detection was 100 μg/kg). Furthermore, the control treatment produced 9 mg/kg of DON.

### 2.4. Pre-Test of DON Degradability by MnP and LiP in Artificial Digestive Juices

**Figure 3:**
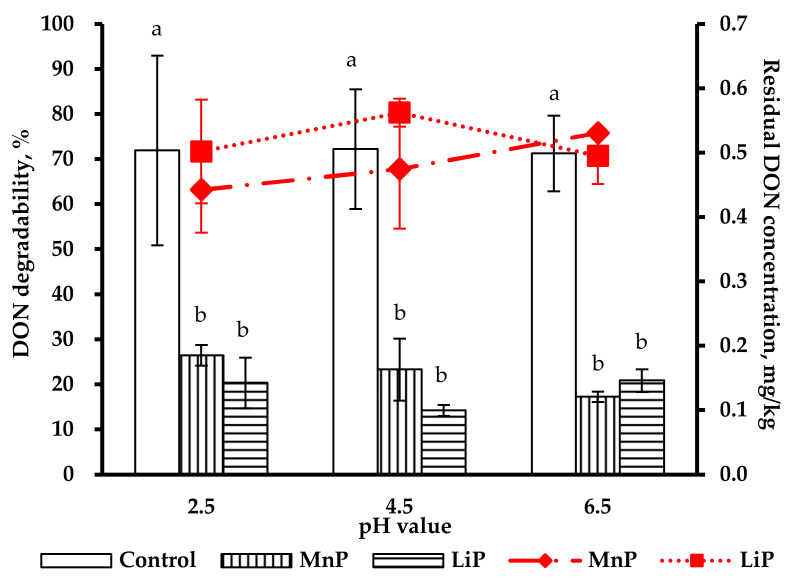
DON degradability (line) and residual concentration (bar) of the Control, MnP (50 U/g) and LiP (50 U/g) in AGJ (pH 2.5 and 4.5) and AIJ (pH 6.5), respectively, at 1 h after peroxidase treatments. ^a,b^ Means for each pH value without the same superscripts differ (*p* < 0.05). AGJ: Artificial gastric juice; AIJ: Artificial intestinal juice; DON: Deoxynivalenol; MnP: Manganese peroxidase. LiP: Lignin peroxidase. Data are expressed as mean ± SD (*n* = 3).

Figure 3 shows the residual concentrations of DON degradability treated with MnP and LiP in artificial digestive juices at various pH (2.5, 4.5, and 6.5) conditions for 1 h. Deoxynivalenol degradabilities of MnP and LiP treatments were 63.2% and 71.7%, respectively, at pH 2.5 in artificial gastric juice (AGJ); under pH 4.5 in AGJ, 67.2% and 80.3% of DON degradations, respectively, were observed after MnP and LiP treatments. In artificial intestinal juice (AIJ) at pH 6.5, as much as 75.8% and 70.7% of DON degradability, respectively, were observed after MnP and LiP treatment. The calculated residual concentrations from the MnP and LiP treatment groups respectively were 0.185 mg/kg and 0.142 mg/kg under pH 2.5 in AGJ, 0.163 mg/kg, and 0.1 mg/kg at pH 4.5 in AGJ, and 0.121 mg/kg and 0.146 mg/kg at pH 6.5 in AIJ. Both the residual concentrations and degradabilities of DON after MnP and LiP treatments were significantly lower than those of the control group (*p* < 0.05), but no difference between MnP and LiP treatment groups was observed. Because LiP treatment showed a better antifungal effect than MnP treatment and no significant difference was observed between MnP and LiP treatments on DON degradability, only the LiP treatment was selected for the subsequent simulation trials in the digestive tracts of both poultry and pigs.

### 2.5. Simulation of Pig Gastrointestinal Tracts in DON Degradability by LiP Treatment

**Figure 4:**
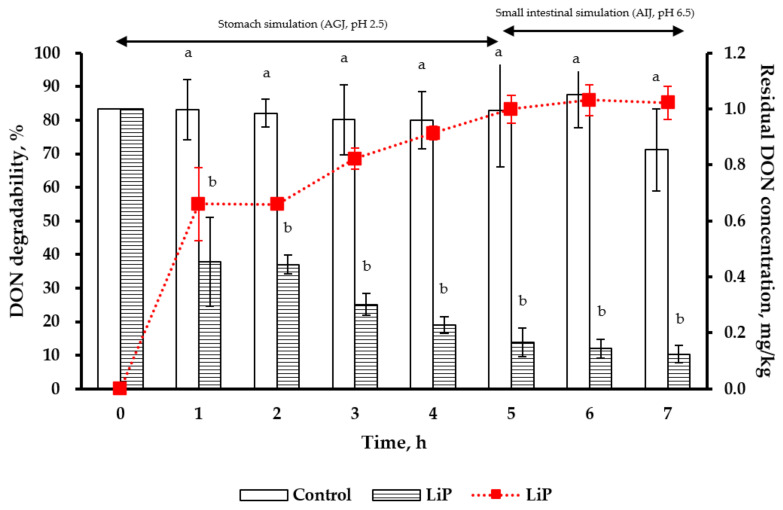
Degradability (line) and residual concentration (bar) of DON after LiP treatment in the simulated pig gastrointestinal tracts (0 to 5 h for stomach simulation and then 6 to 7 h for small intestine simulation). ^a,b^ Means for each pH value without the same superscripts differ (*p* < 0.05). AGJ: Artificial gastric juice; AIJ: Artificial intestinal juice; DON: Deoxynivalenol (1 mg/kg); LiP: Lignin peroxidase (50 U/g). Data are expressed as mean ± SD (*n* = 3).

For the simulation of pig stomach conditions (pH 2.5) for 5 h, the degradability and residual concentration of DON (at 1 mg/kg) were 83.3% and 0.17 mg/kg, respectively, after LiP treatment (Figure 4). Under the simulated pig small intestine conditions (pH 6.5) for 2 h (at the 7th h), the degradability and residual concentration of DON were 85.1% and 0.12 mg/kg, respectively after LiP treatment. Beginning from the first hour throughout the whole simulation period (till 7 h), DON residual concentrations in the LiP-treated groups were all lower than that in the control group (*p* < 0.05).

### 2.6. Simulation of Poultry Gastrointestinal Tracts in DON Degradability by LiP Treatment

**Figure 5:**
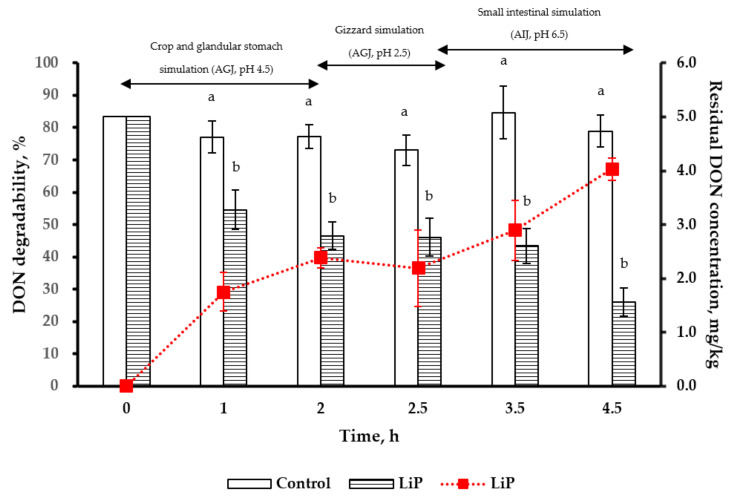
Degradability (line) and residual concentration (bar) of DON in the control and LiP treatment groups under poultry gastrointestinal simulations (0 to 2 h for the crop and glandular stomach, 2 to 2.5 h for the gizzard, and then 2.5 to 4.5 h for the small intestine). ^a,b^ Means for each pH value without the same superscripts differ (*p* < 0.05). AGJ: Artificial gastric juice; AIJ: Artificial intestinal juice; DON: Deoxynivalenol (5 mg/kg); LiP: Lignin peroxidase (50 U/g). Data are expressed as mean ± SD (*n* = 3).

Under the simulated poultry crop and glandular stomach condition (pH 4.5 for 2 h), the degradability and residual concentration of DON (5 mg/kg) after LiP treatment were 39.8% and 2.8 mg/kg, respectively. In the simulated gizzard conditions (pH 2.5 for 0.5 h), the degradability and residual concentration of DON were 36.5% and 2.8 mg/kg (at the 2.5th h), respectively. Under the simulated small intestine condition (pH 6.5 for 2 h), the degradability and residual concentration of DON were 67.2% and 1.6 mg/kg (at the 4.5th h), respectively (Figure 5). The LiP treatment displayed significant degradability for high DON concentration (5 mg/kg) throughout the poultry gastrointestinal simulations. The residual concentrations of DON in the simulated digestive tracts were lower than that in the control group after LiP treatment (*p* < 0.05).

## 3. Discussion

*Fusarium graminearum* is not only a phytopathogenic fungus that induces *Fusarium* Head Blight (FHB) in grains but also produces a highly toxic molecule—DON [26]. There has been an increasing interest in searching for biological antifungal agents to replace synthetic pesticides in recent years. Among natural antifungal compounds, particular interests have been focused on the extracts of plant or agricultural by-products. Akyus and Kirbag [33] suggested that white rot fungi *Pleurotus eryngii* had antimicrobial activity. Schalchli et al. [34,35] also indicated that another species of white-rot fungi *Trametes hirsuta* was able to synthesize antifungal compounds. The current study found that both MnP and LiP treatments had antifungal abilities and LiP was found to be more effective than MnP. The mixtures of MnP and LiP contained H_2_O_2_ and veratryl alcohol, respectively. It has been reported that veratryl alcohol had concentration-independent antifungal activity that could inhibit the growth of phytopathogenic fungi *Sclerotinia sclerotiorum* and *Pythium irregular* [36], but little information is available about the antifungal effects of H_2_O_2_. It is suggested that veratryl alcohol and the peroxidase from the SMS of *F. velutipes* in PDA are the major compounds responsible for the antifungal activity of the LiP treatment. 

The fungal cell wall is comprised of glucan and chitin that play essential roles in several biological functions, such as cell shape, morphogenesis, reproduction, cell-matrix interactions, and physical osmotic protection [37]. The content of chitin is also critical for maintaining the physical strength of cell walls. Many studies have indicated that loss of chitin synthase activity in *F. graminearum* can significantly compromise fungal growth, development, and pathogenicity [38,39]. In the present study, the chitinase activity and GlcNAc content were both increased in MnP and LiP treatments compared to that in the control treatment. In addition, hyphal vesicles became visible inside the cell wall after MnP and LiP treatments, and the hyphae became broken down after LiP treatment (Figure 1). These results strongly suggest that MnP and LiP could enhance chitinase activity and cause degradation or degeneration of hyphae. Nevertheless, further studies are required to disclose the mechanisms behind it.

It is known that white-rot fungi can produce extracellular ligninolytic peroxidases i.e., MnP and LiP, which catalyze the formation of hydroxyl free radicals through oxidative reaction and thus alter the chemical bonds or molecular conformation of their substrates. These ligninolytic peroxidases have extremely low specificity for substrates. While MnP degrades lignin and phenolic compounds by oxidizing the substrate in the presence of H_2_O_2_ and Mn^2+^ [27], LiP degrades various xenobiotic compounds by oxidizing substrates that contain H_2_O_2_ and veratryl alcohol [27]. Moreover, these peroxidases are widely applied in the paper industry and environment protection [40], because they not only degrade lignin but also a wide range of environmental contaminants, such as phenols, polycyclic aromatic hydrocarbons, and aromatic water-pollutants [41]. Deoxynivalenol is a mycotoxin secreted during the secondary metabolism of *F. graminearum,* and is only produced by some fungus lineages [42]. As mentioned, both MnP and LiP treatments were found to inhibit the growth of hyphae and sporulation of *F. graminearum* KR1, which in turn, could minimize DON production after MnP and/or LiP treatments. Therefore, these two SMS-derived peroxidases from *F. velutipes* could potentially be used as biological antifungal agents although LiP appeared more effective than MnP in the present study.

Degradation of mycotoxins by ligninolytic enzymes purified from white-rot fungi has been intensively studied. The crude enzyme extract from SMS of *P. eryngii* [43] and MnP purify from *Pleurotus ostreatus* [44] can both effectively degrade up to 90% of AFB_1_. However, MnP has been verified as being capable of degrading DON effectively [30], but to our best knowledge, no report is available on the DON degradation capacity of LiP. In our preliminary test for DON degradation, the degradability of DON with MnP was 63.2%, 67.8%, and 75.8% at pH 2.5, 4.5, and 6.5, respectively; the best degradability was revealed between pH 4.5 and 6.5. These results were in agreement with the previous studies, in which an optimal pH for MnP activity ranged from 4 to 6 [44,45]. For LiP, its best degradability of DON with 50 U/mL tryptone was 80.3% under pH 4.5 in this study (Figure 3). This is also similar to that reported by S. Rodríguez Couto et al. [45], where the optimal pH was 4.2 at 34 °C Unlike the effects of pH value and temperature, light might not directly affect the biological activities and stabilities of MnP and LiP but there has been no study to date.

In addition to pH value and temperature, small molecule mediators also play an essential role in the peroxidative functions of MnP and LiP. Hydrogen peroxidase and veratryl alcohol are important mediators for intensifying the antifungal reactions and DON degradability by MnP and LiP. In the presence of H_2_O_2_, MnP oxidizes the substrate faster than in other substrates without H_2_O_2_ [46]. In industrial applications, a high concentration of H_2_O_2_ in MnP solution is important to stabilize MnP activity [44]. Christian et al. [47] also indicated that veratryl alcohol enhances the oxidation of LiP on many compounds, including lignin, suggesting that veratryl alcohol acts as a redox mediator.

For our gastrointestinal simulations, DON degradabilities of LiP treatment at 50 U/g were 85.5% and 70.1%, respectively. It was demonstrated that LiP can effectively degrade the nonphenolic derivative—DON in the gastrointestinal simulations of pig and poultry after incubation with feed mixtures for 4.5 h or longer. It is worth mentioning that MnP and LiP could be developed into antifungal agents and DON-degrading feed additives to avoid economic loss of crops. However, currently no study is available on the toxicity or cytotoxicity of MnP, LiP, as well as their metabolites after enzymatic digestion with regard to animals and humans. It has become extremely important to investigate their toxicity before applying these peroxidases as food or feed additives. Besides, the use of suitable carriers (e.g., yeast cell wall) to increase the stability of MnP and LiP during storage should also be carried out in the future. In addition to the potential applications of MnP and LiP, the present study has adequately provided a simplified purification procedure in turning disposed SMS into a valuable by-product from the mushroom industry in an environment-friendly manner. 

## 4. Conclusions

To our best knowledge, this is the first report on the ability of MnP and LiP to inhibit the growth of *F. graminearum* KR1 and the production of DON. The growth inhibition and the chitinase activity of LiP are more effective than that of MnP. Also, effective degradation of DON by LiP treatment in simulated gastrointestinal systems was demonstrated in the present study. Therefore, MnP and LiP are potentially applicable as inhibitory enzymes to the growth and DON production of *F. graminearum* KR1, as well as to degrade existing DON in animal feeds, given that the toxicities of these peroxidases and the resulting metabolites are under strict surveillance. More studies are also required for future applications of MnP and LiP as anti-*Fusarium* or anti-DON feed additives in both food and animal industries.

## 5. Materials and Methods 

### 5.1. Chemicals and Fungal Strain

Deoxynivalenol standard with purity higher than 99% was purchased from Fermentek (Jerusalem, Israel). Acetonitrile and methanol were high-performance liquid chromatography (HPLC) grade purchased from Merck (Darmstadt, Germany). 

Acetic acid, bile salt, chitin, hydrogen peroxidase (H_2_O_2_), manganese sulfate (MnSO_4_), monopotassium phosphate (KH_2_PO_4_), GlcNAc, *p*-dimethylamino benzaldehyde (*p*-DMAB), polyethylene glycol (PEG, molecular weight 8000), pepsin, potassium borate (B_4_K_3_O_3_), sodium acetate (C_2_H_3_NaO_2_), trypsin, Tris-HCl, and veratryl alcohol were purchased from Sigma-Aldrich (Saint Louis, USA). 

Alfa Aesar (Haverhill, MA, USA) supplied 2,6-dimethyoxyphenol (2,6-DMP), tartaric acid, and potassium sodium L-tartrate tetrahydrate. The Czapek solution agar and potato dextrose agar (PDA) were purchased from BD Difco (Franklin Lakes, NJ, USA).

The commercial pig and poultry feed used in the in vitro procedure were provided by Farmwealth (Tainan, Taiwan). *F. graminearum* KR1, isolated from the wheat in Taiwan and maintained in PDA at 4 °C, was provided by Dr. C. L. Wang, Department of Plant Pathology, National Chung Hsing University, Taichung, Taiwan. 

### 5.2. Extraction and Analyses of MnP and LiP

The SMS of *F. velutipes* used to extract MnP and LiP were obtained from a mushroom farm (Dai Yang farm) in Wufeng district (Taichung, Taiwan). The substrate of *F. velutipes* consisting of sawdust, rice bran, wheat bran, and corn flour, was collected after the harvest of golden needle mushroom. The procedures for the extraction and analysis of MnP and LiP were based on previous studies [48,49,50], with some modifications. Briefly, to 120 g SMS of *F. velutipes,* 600 mL of 1 M sodium acetate buffer (pH 3) and 0.25 M tartaric acid buffer (pH 4.5) were added for the extractions of MnP and LiP, respectively. After standing at room temperature for 24 h, the enzymes were extracted as follows. Samples were centrifuged at 10,000× *g* for 10 min before the collection of supernatants through filter paper (Whatman#1, Whatman, Maidstone, England). For MnP, the reaction mixture was 2.5 mL and consisted of 0.5 mL extracted MnP filtrate, 0.75 mL water, 0.5 mL of 5 mM 2,6-DMP, 0.25 mL of 0.01 M MnSO_4_, 0.25 mL 0.1 mM of H_2_O_2_, and 0.25 mL of 0.1 M sodium acetate buffer (pH 4.5) [48,49]. For LiP, the reaction mixture was 2.5 mL and consisted of 0.75 mL water, 0.5 mL extracted LiP filtrate, 0.5 mL of 10 mM veratryl alcohol, 0.25 mL of 0.5 M H_2_O_2_, and 0.25 mL of 0.25 M tartrate buffer (pH 3) [49,50]. The reaction mixtures were placed in a glass cryotube, stored at −20 °C for freeze-dry preparation, and then connected to a freeze dryer (FD-12-2S-GP, Kingmech Co. New Taipei City, Taiwan) for freeze-drying based on the manufacturer’s instruction. The dried powders of MnP and LiP were stored at −20 °C until use. 

The activities of MnP and LiP were evaluated using spectrometry (SmartSpec Plus, Bio-Rad, Hercules, CA, USA) at 469 nm and 310 nm, respectively. For enzyme activities, extinction coefficients of 27500 M^−1^ cm^−1^ for MnP and 9300 M^−1^ cm^−1^ for LiP were used and estimated according to the following equations:

*Manganese peroxidase activity* (U/mL) = *ΔA/min* × 2.5 × 10^6^ × *D*/27500 M^−1^ cm^−1^ (1)

*Lignin peroxidase activity* (U/mL) = *ΔA/min* × 2.5 × 10^6^ × *D*/9300 M^−1^ cm^−1^ (2)

where *ΔA/min* = Increase in absorbance for 1 min at 469 nm for MnP or 310 nm for LiP

*D* = Sample dilution 

The calculated activities of MnP and LiP were respectively 67.8 ± 8.1 U/mL and 1914 ± 37 U/mL in the reaction mixtures, and 237 ± 2.6 U/g and 2201 ± 15.6 U/g in the powders. The experiment was carried out with three replicates per treatment.

### 5.3. Antifungal Activity Assays

The methods to determine antifungal activities of MnP and LiP were according to Cho et al. [51] In the control treatment, a 0.5 cm colony of *F. graminearum* KR1 was cultivated in PDA at 25 °C in the dark for 7 days. In the treatment groups, the PDA was autoclaved (121 °C, 15 min) and cooled to approximately 50 °C. Then, MnP and LiP solutions were added into PDA with the concentration adjusted to 50 U/mL. A 0.5 cm colony of *F. graminearum* KR1 was inoculated at the center of each PDA containing MnP and LiP at 25 °C in darkness for 7 days, and the growth of colonies was observed. Eight repeats of the experiment were conducted for each treatment group. For assessing the growth inhibition rate, the colony diameter was measured regularly by day. The growth inhibition rate of the mycelium was calculated as follows:

*Growth inhibition rate (%)* = (1 − *diameter of mycelium in the MnP-and LiP-treated medium/diameter of mycelium in the non-treated medium)* × 100 (3)

### 5.4. Determination of GlcNAc Content, Chitinase Activity, and Hyphae Morphology 

The hyphal preparation, hyphal morphology observation, and analysis of GlcNAc content and chitinase activity were conducted according to methods described by Shi et al. [52]. Briefly, the *F. graminearum* KR1 pathogen was grown in PDA at 25 °C for 7 days, and the 10 mm mycelium was inoculated into 500 mL flasks containing 250 mL of sterilized Czapek solution agar and cultured in flasks with 140 rpm at 25 °C for 72 h. Then, MnP and LiP were added to a final concentration of 50 U/mL and the mycelium growth was observed 1, 3, 6, and 12 h after treatment. The effects of MnP and LiP on the hyphal morphology were observed using a light microscope (E100, Nikon, Tokyo, Japan). The hyphal materials (0.4 g dry weight) were harvested and homogenized in 2 m L of Tris-HCl (0.05 M, pH 7.5). The homogenate was centrifuged at 15,000× *g* for 10 min at 4 °C, and the supernatant was used to determine GlcNAc content and chitinase activity. For determining GlcNAc content, 0.2 mL of supernatant liquid was treated with 0.1 mL of 0.8 M potassium borate solution, and the mixture was placed in a boiling water bath for 3 min. After cooling down, 3 mL of 1% *p*-DMAB was added, and the samples were kept at 36 °C for 20 min, and then immediately analyzed by spectrophotometer at 544 nm. For determining chitinase activity, gelatin form chitin (0.2 mL of *N*-acetylation product of chitosan) was added to 0.3 mL of supernatant liquid, and the mixture was kept at 37 °C for 1 h and inactivated in boiling water for 5 min. After centrifugation at 5000× *g* for 10 min, 0.2 mL of supernatant liquid was kept in a boiling water bath for 3 min. After cooling down, 0.1 mL of 0.8 M potassium borate solution and 3 mL of 1% *p*-DMAB were added. After incubation at 36 °C for 20 min, the sample was immediately analyzed by spectrophotometer at 544 nm. Different GlcNAc concentrations were used to establish the standard curves for assessing GlcNAc content and chitinase activity. Three repeats were carried out in each treatment.

### 5.5. Preparation of Artificial Digestive Juices

The AGJ and AIJ were prepared according to Tso et al. [53] and Wang et al. [15]. For the preparation of AGJ, sodium chloride (2 g) and pepsin (3.2 g) were dissolved in sufficient water. Five mL of 36.5% HCl was added into the solution and aliquoted to 1000 mL with water to obtain the AGJ followed by adjusting the pH values to 2.5 and 4.5 with 0.1 M NaOH. For the preparation of AIJ, KH_2_PO_4_ (6.8 g) was dissolved in 500 mL water, and the pH value was adjusted to 6.8 with 0.1 M NaOH. Ten grams of trypsin were dissolved in water to mix with the KH_2_PO_4_ solution. Then, 3 g of porcine bile salt was added, aliquoted to 1000 mL, and adjusted pH 6.5 with 36.5% HCl. Both AGJ and AIJ were kept at 4 °C before use.

### 5.6. A Pre-Test for DON Degradability of MnP and LiP

DON standard solution was added to the feed for adjusting DON concentration to 0.5 mg/kg. One hundred and eighty grams of poultry feed containing 0.5 mg/kg DON was premixed with 50 U/g of MnP and LiP powder, respectively. Sixty grams of peroxidase-treated feed was added to a 500 mL centrifuge tube and mixed with AGJ (pH 2.5 and then 4.5) or AIJ (pH 6.5) at a 1:3 (*w*/*v*) ratio. The pH values of the resulting solutions were adjusted to 2.5, 4.5, or 6.5 with 36.5% HCl or 0.1 M NaOH before incubating in a shaking incubator (40.0 ± 5 rpm) at 40.0 ± 1 °C for 1 h. From each treatment group, 20 mL of AGJ or AIJ mixture was collected and centrifuged with 5000× *g* for 10 min at 4 °C; then 10 mL of centrifuged AGJ or AIJ supernatant was added to DON extraction solution for the analysis of residual DON concentrations. The experiment was carried out with three replicates per treatment.

### 5.7. Simulations of Pig and Poultry Gastrointestinal Tracts with LiP Treatment

Similar to the pre-test procedure, the simulation conditions for the gastrointestinal tracts of pig and poultry were based on the procedures reported in Tso et al. [53]. Different DON concentrations for pig (1 mg/kg) and poultry (5 mg/kg) feeds were used based on a report from the China Hygienic Standard for Feed (GB13078-2017) and FDA regulations [54]. The DON standard was added to the commercial pig and poultry feeds to adjust the desired DON concentration. 

As described previously, 180 g of pig and poultry feeds, each with DON concentration of 0.5 mg/kg DON, were premixed with LiP powder (50 U/g). The mixture (60 g) was added to a 500 mL centrifuge tube to mix with AGJ at a 1:3 (*w*/*v*) ratio; the pH was then adjusted to target value and cultured in an incubator at 40 °C with constant shaking (40 rpm). Twenty mL of AGJ or AIJ mixtures was collected hourly and centrifuged for DON extraction. Similarly, 10 mL of centrifuged AGJ or AIJ supernatant was directly added to the DON extraction solution.

For the stimulation in the pig stomach, the pH 2.5 of AGJ was incubated for 5 h. For the stimulated poultry crop and glandular stomach, the pH 4.5 of AGJ was first incubated for 2 h and then the AGJ was adjusted to pH 2.5 to mimic gizzard conditions (for 30 min). 

Before pig and poultry small intestine simulations, treatment mixtures were also centrifuged at 5000× *g* for 10 min (4 °C) and filtered to remove AGJ; the residual feed (pellet) was stored under −20 °C for further determination. The samples of AGJ from each treatment were immediately extracted and analyzed to determine the DON concentrations and LiP activity. After this process, the DON standard was added to the feed samples in order to adjust DON concentration and LiP activity to the desired conditions of AGJ for the small intestinal simulations; thereafter, the AIJ was added (1:3, *w*/*v*) and the pH was adjusted (6.5) followed by a 2 h-incubation to mimic the small intestine condition of pigs and poultry. The experiment was carried out with three replicates per treatment.

### 5.8. DON Extraction and Assay

The DON analysis was slightly modified according to methods described in Tso et al. [53]. For extracting DON from the solid samples (PDA and feed), 5 g of solid sample was mixed with 20 mL of DON extraction solution (1 g PEG/20 mL water) in a 250 mL quartz cup. The mixture was blended by a homogenizer (PT-MR 3000, Brinkmann, Germany) at 40,000 rpm for 5 min, and then was filtered through filter paper (Whatman#1, Whatman, England) and a glass fiber filter (Vicam#31955, Vicam, Milford, MA, USA). For extracting DON from the artificial digestive juices (AGJ and AIJ), 10 mL of artificial digestive juice was mixed with 10 mL of DON extraction solution (1 g PEG/10 mL water) in a 50 mL centrifuge tube and then vortexed for 2 min (Vortex-Genie 2, Scientific Industries, Bohemia, NY, USA) before filtering through a glass microfiber filter. The procedures for DON purification and HPLC analysis were the same as for liquid samples. 

For purifying DON, 2 mL of filtered extract was completely passed through the DONTest^TM^ affinity column (Vicam, Milford, MA, USA). Subsequently, the affinity column was washed with 5 mL water, eluting the affinity column by passing 2 mL methanol through the column and collecting all the sample eluate in a glass cuvette. The eluate was dried by a nitrogen concentrator (Pierce React-Therm III^TM^, Thermo Fisher Scientific, Waltham, MA, USA), resuspended in 2 mL acetonitrile-water (10:90, *v*/*v*), mixed well by vortexing, and then filtered with a syringe filter (pore size: 0.45 μm; Micron Separations, Westborough, MA, USA) for HPLC analysis. For analysis, 50 μL of the filtered sample was injected into an HPLC pump (L-2130, Hitachi, Japan) by using an auto-sampler (L-2200, Hitachi, Tokyo, Japan) with the ultraviolet (UV) detector at 220 nm. The mobile phase (acetonitrile-water, 10:90, *v*/*v*) was filtered through a 0.45 μm filter membrane (Micron Separations, Westborough, MA, USA), degassed, and analyzed at the flow rate of 1 mL/min. The HPLC column used was a Mightysil^TM^ RP-C18 column (4.6 × 250 mm, 5 μm, Kanto Chemical, Tokyo, Japan).

### 5.9. Statistical Analysis

All data in this study were analyzed with Analysis of Variance (ANOVA), and the General Linear Model (GLM) procedure in the PC-SAS^®^ version 9.2 statistical software (SAS Institute, 2008) was used. Tukey’s test was used to determine differences between individual means at a significance level *p* < 0.05, unless otherwise stated. All the experimental data were expressed as mean ± SD.

## Figures and Tables

**Table 1:** Growth inhibition of *F. graminearum* KR1 by manganese peroxidase (MnP) and lignin peroxidase (LiP) treatments ^1^.

Item	Incubation Time, d						
	1	2	3	4	5	6	7
Diameter, cm							
Control ^1^	2.86 ± 0.73	4.78 ± 1.04	5.28 ± 1.16	6.50 ± 1.91	6.85 ± 1.99	7.78 ± 2.43	9.50 ± 0.40
MnP	2.90 ± 0.38	4.70 ± 0.63	5.28 ± 0.73	6.12 ± 1.30	6.34 ± 1.12	6.62 ± 1.24	7.28 ± 1.25
LiP	1.54 ± 0.36	2.02 ± 0.53	2.30 ± 0.40	2.28 ± 0.12	2.26 ± 0.60	2.45 ± 0.54	2.41 ± 0.29
Inhibition rate, %							
MnP	−5.26 ± 18.7 ^b^	−0.50 ± 12.3 ^b^	−2.11 ± 12.8 ^b^	3.30 ± 11.3 ^b^	3.97 ± 16.4 ^b^	9.44 ± 23.6 ^b^	23.7 ± 10.2 ^b^
LiP	45.2 ± 8.67 ^a^	57.8 ± 4.88 ^a^	55.9 ± 3.54 ^a^	62.1 ± 11.4 ^a^	66.6 ± 4.77 ^a^	67.3 ± 5.10 ^a^	74.7 ± 2.23 ^a^

^1^ Control: only potato dextrose agar (PDA); MnP: PDA with 50 U/mL of manganese peroxidase; LiP: PDA with 50 U/mL of lignin peroxidase. ^a,b^ Means for each time point of incubation without the same superscripts differ (*p* < 0.05). Data are expressed as mean ± SD (*n* = 8).

**Table 2:** Chitinase activity in the hyphae and *N*-acetyl-d-glucosamine (GlcNAc) content of *F. graminearum* KR1 in Czapek solution agar.

Item	Incubation Time, h			
	1	3	6	12
Chitinase activity, GlcNAc mg/g fresh weight/h				
Control ^1^	0.000 ± 0.000 ^b^	0.012 ± 0.001 ^c^	0.033 ± 0.001 ^c^	0.075 ± 0.003 ^b^
MnP	0.014 ± 0.002 ^a^	0.066 ± 0.011 ^b^	0.095 ± 0.006 ^b^	0.117 ± 0.006 ^b^
LiP	0.014 ± 0.003 ^a^	0.120 ± 0.015 ^a^	0.179 ± 0.028 ^a^	0.231 ± 0.043 ^a^
GlcNAc content, mg/g fresh weight				
Control ^1^	0.000 ± 0.000 ^b^	0.011 ± 0.000 ^c^	0.040 ± 0.002 ^c^	0.092 ± 0.002 ^b^
MnP	0.020 ± 0.002 ^a^	0.072 ± 0.012 ^b^	0.084 ± 0.009 ^b^	0.093 ± 0.017 ^b^
LiP	0.019 ± 0.003 ^a^	0.114 ± 0.010 ^a^	0.174 ± 0.012 ^a^	0.286 ± 0.021 ^a^

^1^ Control: only Czapek solution agar (CZ); MnP: CZ with 50 U/mL of manganese peroxidase; LiP: CZ with 50 U/mL of lignin peroxidase. ^a–c^ Means from each time point of incubation without the same superscripts differ (*p* < 0.05). Data are expressed as mean ± SD (*n* = 3).

## Data Availability

Data available in a publicly accessible repository.
